# VMP1 forms a Ca^2+^ release channel essential for postnatal heartbeat

**DOI:** 10.1126/sciadv.adz0706

**Published:** 2026-06-12

**Authors:** Yuying Ma, Qiguang Li, Yuting Jia, Bo Hao, Jiahe Li, Ziyi Zhang, Na Yin, Sui Fang, Yao Wang, Zhifang Wu, Zhaobing Gao, Bingqing Xia, Min Peng

**Affiliations:** ^1^ State Key Laboratory of Molecular Oncology, Institute for Immunology, Beijing Key Laboratory of Immunological Research of Allergy, School of Basic Medical Sciences, Tsinghua Medicine, Tsinghua University, Beijing, China.; ^2^ SXMU-Tsinghua Collaborative Innovation Center for Frontier Medicine, Shanxi Medical University, Taiyuan, Shanxi Province, China.; ^3^ Tsinghua-Peking Center for Life Sciences, Beijing, China.; ^4^ State Key Laboratory of Drug Research, Shanghai Institute of Materia Medica, Chinese Academy of Sciences, Shanghai, China.; ^5^ University of Chinese Academy of Sciences, Beijing, China.; ^6^ Department of Nuclear Medicine, First Hospital of Shanxi Medical University, Shanxi Medical University, Taiyuan, China.; ^7^ Collaborative Innovation Center for Molecular Imaging of Precision Medicine, Shanxi Medical University, Taiyuan, China.; ^8^ School of Forensic Medicine, Shanxi Medical University, Taiyuan, China.; ^9^ Key Laboratory of Forensic Medicine in Shanxi Province, Shanxi Medical University, Taiyuan, China.

## Abstract

Normal heart contraction requires synchronized calcium ion (Ca^2+^) release from the sarcoplasmic reticulum (SR), traditionally attributed to ryanodine receptor 2 (RyR2). Here, we identify vacuole membrane protein 1 (VMP1) as a previously unrecognized SR Ca^2+^ release channel essential for postnatal cardiac function. VMP1 expression is up-regulated in cardiomyocytes after birth, and its genetic deletion causes severe arrhythmias, dilated cardiomyopathy, and sudden cardiac death. Mechanistically, VMP1 loss results in increased SR Ca^2+^ content and aberrant cardiac action potentials. Single-channel electrophysiology reveals that VMP1 forms a Ca^2+^-regulated Ca^2+^ channel, which senses luminal Ca^2+^ via aspartic acid 272. Notably, VMP1 expression is elevated in human heart failure, suggesting a pathophysiological role. These findings establish VMP1 as a critical component of the cardiac Ca^2+^ release machinery and uncover its involvement in heart failure.

## Teaser

VMP1 is a key SR Ca^2+^ release channel essential for cardiac function, with involvement in heart failure.

## INTRODUCTION

The rhythmic contraction of the heart depends on the highly coordinated release and reuptake of calcium ion (Ca^2+^) by the sarcoplasmic reticulum (SR) in cardiomyocytes (*1*). During each heartbeat, action potentials (APs) trigger a small Ca^2+^ influx through L-type calcium channels, which subsequently activates ryanodine receptor 2 (RyR2) to mediate a large-scale Ca^2+^ release from the SR, a process known as Ca^2+^-induced Ca^2+^ release (CICR) (*2*, *3*). The released Ca^2+^ binds to troponin C, enabling myofilament cross-linking and heart contraction. RyR2, the principal SR Ca^2+^ release channel, is critical for this process, and its dysfunction is strongly associated with arrhythmias and heart failure (*4*–*8*). During cardiac relaxation, Ca^2+^ is removed from the cytosol via extrusion through the sarcolemma and reuptake into the SR (*9*). Extrusion occurs through Na^+^/Ca^2+^ exchangers, while SR reuptake is mediated by the activation of endoplasmic reticulum (ER)/SR Ca^2+^ adenosine triphosphatase (SERCA) (*9*).

While SERCA-mediated Ca^2+^ pumping is the sole mechanism of SR Ca^2+^ uptake, RyR2 alone may not fully explain SR Ca^2+^ release in various physiological and pathological contexts. The functional diversity of Ca^2+^ signaling in development and disease implies the existence of additional ER/SR Ca^2+^ release channels (*10*, *11*), yet their identities remain elusive. Resolving this question is essential for advancing our understanding of cardiac excitation-contraction coupling (ECC), arrhythmogenesis, and heart failure, a leading cause of morbidity and mortality worldwide.

Vacuole membrane protein 1 (VMP1) is an ER-resident transmembrane protein initially identified as a regulator of autophagy (*12*, *13*). Later studies revealed that VMP1 also functions as a lipid scramblase to regulate metabolism (*14*–*16*). In addition, VMP1 plays an essential role in viral infections, including severe acute respiratory syndrome coronavirus 2 (*17*). However, the biochemical nature of VMP1 remains to be determined. Recently, VMP1 has been shown to be involved in Ca^2+^ release from the ER in T cells (*18*), but whether VMP1 forms a Ca^2+^ channel remains to be determined.

Considering that SR is a specialized form of the ER and SR Ca^2+^ release is critical for heart contraction, we hypothesized that VMP1 might play a role in heart contraction. In this study, we show that VMP1 forms a SR Ca^2+^ release channel indispensable for postnatal heartbeat, addressing a long-standing gap in the field and providing insights into cardiac physiology and disease mechanisms.

## RESULTS

### Induced VMP1 expression in the mouse heart after birth

VMP1 is a transmembrane protein that resides in the ER (*12*, *13*) and has recently been implicated in ER Ca^2+^ release in immune cells (*18*). However, whether VMP1 facilitates Ca^2+^ release in other cell types and whether it functions as a Ca^2+^ channel remain unknown. Given that SR is a specialized form of the ER in muscle cells, we explored the role of VMP1 in SR Ca^2+^ regulation and cardiac function.

**Fig. 1.**
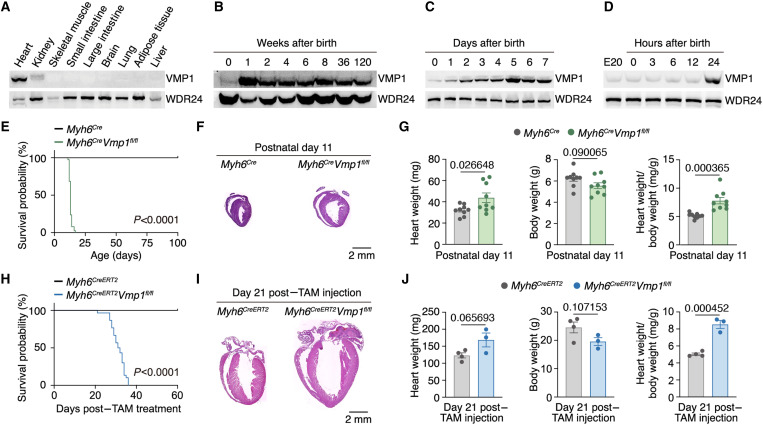
VMP1 is up-regulated in the postnatal heart, and its deficiency causes sudden cardiac death in mice. (**A**) VMP1 expression in the indicated tissues of adult (8-week-old) C57BL/6 mice was examined by immunoblotting, with WDR24 used as the loading control. Representative blots from five independent experiments are shown. (**B** to **D**) VMP1 expression in the hearts of C57BL/6 mice at the indicated developmental stages was examined by immunoblotting. E20 refers to embryonic day 20. Representative blots from three independent experiments are shown. (**E**) Survival curve of control and *Myh6^Cre^Vmp1^fl/fl^* mice (*n* = 38 mice per group). (**F**) Representative H&E staining of longitudinal heart sections from control and *Myh6^Cre^Vmp1^fl/fl^* mice at postnatal day 11. (**G**) Heart weight, body weight, and heart weight/body weight ratio in control and *Myh6^Cre^Vmp1^fl/fl^* mice at postnatal day 11 (*n* = 9 mice per group). (**H**) Survival curve of control (*n* = 55) and *Myh6^CreERT2^Vmp1^fl/fl^* mice (*n* = 30) following tamoxifen (TAM) injection. (**I**) Representative H&E staining of longitudinal heart sections from control and *Myh6^CreERT2^Vmp1^fl/fl^* mice at 21 days post–tamoxifen injection. (**J**) Heart weight, body weight, and heart weight/body weight ratio in control (*n* = 4) and *Myh6^CreERT2^Vmp1^fl/fl^* mice (*n* = 3) at 21 days post–tamoxifen injection. Data represent the means ± SEM. *n* indicates the number of mice. Exact *P* values were determined by a log-rank (Mantel-Cox) test in (E) and (H) and a two-tailed unpaired Student’s *t* test in (G) and (J).

To begin, we assessed VMP1 protein expression across various organs and tissues in adult C57BL/6 mice. VMP1 expression was highest in the heart, moderate in the kidney, and low to undetectable in skeletal muscle, intestines, brain, lung, adipose tissue, and liver (Fig. 1A). WDR24 was used as the loading control because specialized actin is expressed in cardiomyocytes.

Given the developmental regulation of cardiac function (*19*, *20*), we next analyzed VMP1 expression in heart tissues at different postnatal stages. While VMP1 was nearly undetectable in the hearts of neonatal mice at birth, its expression became relatively stable from 1 to 120 weeks after birth (Fig. 1B). To pinpoint the timing of VMP1 induction, we conducted a detailed time-course analysis. VMP1 expression began increasing on postnatal day 1, continued to rise until day 5, and then stabilized (Fig. 1C). Further analysis revealed that VMP1 expression was low in the fetal heart at embryonic day 20 (E20), remained minimal 12 hours after birth, and was rapidly up-regulated by 24 hours after birth (Fig. 1D).

After establishing that VMP1 is highly enriched in the adult heart, we further examined its subcellular localization in cardiomyocytes. Immunofluorescence analysis showed that VMP1 exhibited a reticular distribution that overlapped with SERCA2-marked SR/ER structures (fig. S1A), with fluorescence intensity analysis confirming strong colocalization (fig. S1B), indicating that VMP1 is predominantly localized to the SR. Together, these findings reveal that VMP1 is highly expressed in the adult heart compared to other organs and demonstrate that its expression is induced shortly after birth.

### VMP1 deficiency causes arrhythmias and sudden death in mice

To investigate the role of VMP1 in cardiac function, we conditionally deleted *Vmp1* specifically in cardiomyocytes using the *Myh6^Cre^* mouse strain (fig. S2A) (*21*). The *Myh6^Cre^* mice expresses Cre recombinase specifically in cardiomyocytes under the control of the *Myh6* (myosin heavy chain 6) promoter, allowing constitutive gene deletion in cardiomyocytes from the early embryonic stage. Genomic DNA analysis confirmed the presence of floxed *Vmp1* alleles in both tail and heart tissues of *Myh6^Cre^Vmp1^fl/fl^* mice (fig. S2B). Deletion bands for *Vmp1* were detected exclusively in the heart, confirming cardiac-specific *Vmp1* ablation (fig. S2C). However, no live *Myh6^Cre^Vmp1^fl/fl^* mice were recovered by weaning age (3 weeks postnatal). Daily monitoring revealed that all *Myh6^Cre^Vmp1^fl/fl^* mice consistently died between postnatal days 12 and 14 (Fig. 1E). Histological analysis of these mice showed features consistent with dilated cardiomyopathy (Fig. 1, F and G, and fig. S2D).

Given the early lethality of *Myh6^Cre^Vmp1^fl/fl^* neonates, we next used the *Myh6^CreERT2^* system to inducibly delete *Vmp1* in adult cardiomyocytes. Eight-week-old *Myh6^CreERT2^* mice were administered tamoxifen (four doses, every other day) to achieve *Vmp1* deletion (fig. S2E). VMP1 expression was notably reduced in isolated cardiomyocytes from *Myh6^CreERT2^Vmp1^fl/fl^* mice compared to controls (fig. S2F), confirming efficient gene deletion. Notably, all *Myh6^CreERT2^Vmp1^fl/fl^* mice succumbed to sudden death within 5 weeks following tamoxifen treatment (Fig. 1H). Histological examination revealed hallmark features of dilated cardiomyopathy in *Myh6^CreERT2^Vmp1^fl/fl^* mice, which were absent in tamoxifen-treated controls (Fig. 1, I and J, and fig. S2G).

Together, these findings demonstrate that while VMP1 is dispensable for cardiac function during fetal and early neonatal stages (before 12 days postnatal), it becomes essential for maintaining cardiac function starting around 12 days after birth. Loss of VMP1 in cardiomyocytes leads to dilated cardiomyopathy and sudden death, underscoring its critical role in postnatal heart function.

### Cardiac dysfunction in the absence of VMP1

**Fig. 2.**
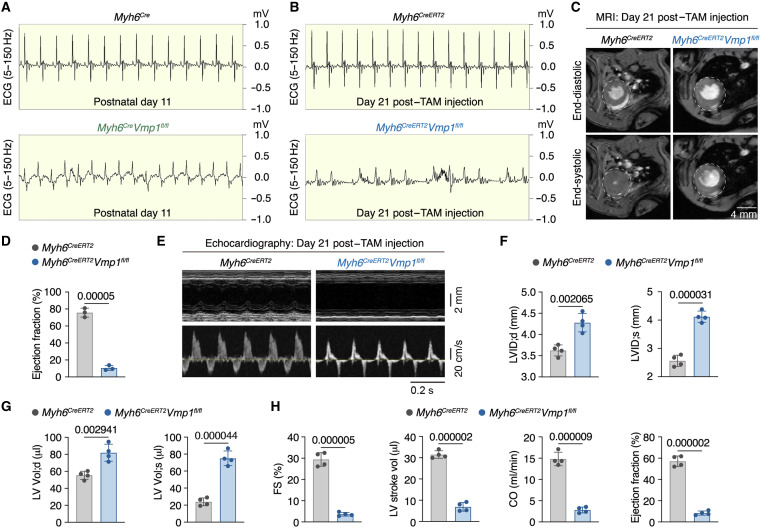
VMP1 deficiency causes severe arrhythmias and heart failure. (**A**) Representative ECG recordings from control and *Myh6^Cre^Vmp1^fl/fl^* mice at postnatal day 11. (**B**) Representative ECG recordings from control and *Myh6^CreERT2^Vmp1^fl/fl^* mice at 21 days post–tamoxifen injection. (**C**) Representative MRI images of the short axis of the heart in control and *Myh6^CreERT2^Vmp1^fl/fl^* mice at 21 days post–tamoxifen injection. The heart is outlined with a white dashed line. (**D**) Percentage of ejection fraction measured by MRI (*n* = 3 mice per group). (**E**) Echocardiographic analysis of control and *Myh6^CreERT2^Vmp1^fl/fl^* mice at 21 days post–tamoxifen injection. Top panel: M-mode parasternal short-axis view at the midventricular level, showing the end-diastolic and end-systolic dimensions of the left ventricle. Bottom panel: Pulsed Doppler imaging at the mitral valve inflow, displaying the early-diastolic and late-diastolic mitral blood flow velocities. (**F** to **H**) Scatterplots of echocardiographic analysis showing left ventricular end-diastolic internal diameter (LVID;d) and end-systolic internal diameter (LVID;s) (F), diastolic and systolic left ventricular volumes (G), and percentage of fractional shortening (FS), stroke volume, cardiac output (CO), and ejection fraction of the left ventricle (H). Data were obtained from transthoracic M-mode tracings of control and *Myh6^CreERT2^Vmp1^fl/fl^* mice at 21 days post–tamoxifen injection (*n* = 4 mice per group). Data represent the means ± SEM. *n* indicates the number of mice. Exact *P* values were determined by a two-tailed unpaired Student’s *t* test.

We next evaluated cardiac function in VMP1-deficient mice. Given that *Myh6^Cre^Vmp1^fl/fl^* mice began to die after postnatal day 12 (Fig. 1E), we performed electrocardiogram (ECG) recordings before this time point. Ten-day-old *Myh6^Cre^Vmp1^fl/fl^* mice did not exhibit obvious cardiac arrhythmias (fig. S3A). However, by postnatal day 11, these mice displayed severe cardiac arrhythmias (Fig. 2A and fig. S3A), providing an explanation for their sudden death between postnatal days 12 and 14 (Fig. 1E). Similarly, tamoxifen-treated *Myh6^CreERT2^Vmp1^fl/fl^* mice developed severe arrhythmias 21 days posttreatment (Fig. 2B). These results indicate that VMP1 is critical for normal ECC in cardiomyocytes starting at postnatal day 11.

Consistent with these observations, the cardiac function assessed via magnetic resonance imaging (MRI) revealed a markedly increased end-systolic internal volume of the left ventricle (LV) (Fig. 2C), resulting in a significant reduction in ejection fraction (Fig. 2D). Echocardiographic evaluation further corroborated the MRI findings. In tamoxifen-treated *Myh6^CreERT2^Vmp1^fl/fl^* mice, echocardiography demonstrated significant increases in the end-diastolic and end-systolic internal diameters and volumes of the left ventricle (Fig. 2, E to G). These changes led to marked reductions in the short-axis contraction fraction, stroke volume, cardiac output, and ejection fraction (Fig. 2H). Together, these functional assessments demonstrate that VMP1 is essential for maintaining normal cardiac function in mice beginning on postnatal day 11.

### Haplosufficiency of VMP1 in cardiac function

RyR2 has been reported to exhibit haploinsufficiency in cardiomyocytes (*22*). The notable phenotype observed in VMP1-deficient hearts prompted us to investigate whether VMP1 function is gene dose–dependent. Deletion of one allele of *Vmp1* using *Myh6^Cre^* did not affect the development or survival of *Myh6^Cre^Vmp1^fl/+^* mice (fig. S3B). Histological analysis revealed normal heart weight in these mice (fig. S3, C and D), and no cardiac arrhythmias were detected (fig. S2, E and F). These findings demonstrate that VMP1 does not exhibit haploinsufficiency in the heart.

### Increased SR Ca^2+^ content and altered excitability in VMP1-deficient cardiomyocytes

VMP1 has been implicated in ER Ca^2+^ release in T cells (*18*). Given the importance of SR Ca^2+^ handling in cardiac ECC, we examined the SR structure and Ca^2+^ storage in VMP1-deficient cardiomyocytes. Confocal imaging of SERCA2 and RyR2 showed no detectable differences in SR marker distribution or sarcomeric organization between VMP1-deficient and wild-type cardiomyocytes (fig. S4, A and B). Both genotypes displayed the characteristic striated SR-T-tubule pattern of mature ventricular myocytes, indicating that VMP1 deficiency does not disrupt global SR architecture.

**Fig. 3.**
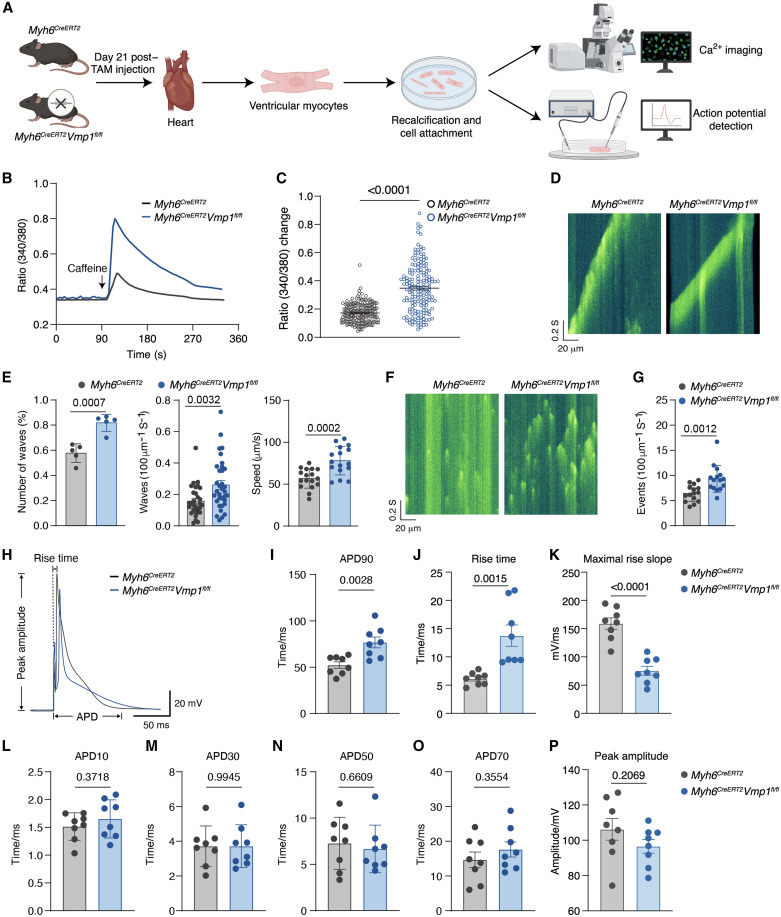
VMP1-deficient cardiomyocytes exhibit increased SR Ca^2+^ content and aberrant AP. (**A**) Schematic diagram of cardiomyocyte isolation, calcium imaging, and AP detection. (**B**) Representative average traces of Ca^2+^ imaging of cardiomyocytes isolated from control and *Myh6^CreERT2^Vmp1^fl/fl^* mice at 21 days post–tamoxifen injection. (**C**) Changes in the ratio of fluorescence intensities (340/380) in cardiomyocytes from control and *Myh6^CreERT2^Vmp1^fl/fl^* mice at 21 days post–tamoxifen injection in response to caffeine stimulation (*n* > 100 cells). (**D**) Representative fluorescence surface plot of spontaneous Ca^2+^ waves of cardiomyocytes isolated from control and *Myh6^CreERT2^Vmp1^fl/fl^* mice. (**E**) Quantification of the percentage (*n* = 5), occurrence frequency (*n* ≥ 30), and speed (*n* ≥ 16) of spontaneous Ca^2+^ waves in cardiomyocytes isolated from control and *Myh6^CreERT2^Vmp1^fl/fl^* mice. (**F**) Representative fluorescence surface plot of spontaneous Ca^2+^ sparks of cardiomyocytes isolated from control and *Myh6^CreERT2^Vmp1^fl/fl^* mice. (**G**) Quantification of the Ca^2+^ spark of cardiomyocytes isolated from control and *Myh6^CreERT2^Vmp1^fl/fl^* mice (n ≥ 15). (**H**) Representative AP traces of cardiomyocytes from control and *Myh6^CreERT2^Vmp1^fl/fl^* mice at 21 days post–tamoxifen injection. (**I **to **K**) Comparison of AP parameters in cardiomyocytes from control and *Myh6^CreERT2^Vmp1^fl/fl^* mice at 21 days post–tamoxifen injection (*n* = 8 mice per group), including AP durations at 90% repolarization (APD90) (I), as well as rise time (J) and maximal rise slope (K). (**L** to **P**) Comparison of other AP parameters in cardiomyocytes from control and *Myh6^CreERT2^Vmp1^fl/fl^* mice at 21 days post–tamoxifen injection (*n* = 8 mice per group), including AP durations at 10, 30, 50, and 70% (APD10, APD30, APD50, and APD70, respectively), as well as peak amplitude. Data represent the means ± SEM. *n* indicates the number of cells (C and E) or mice (G and I to P). Exact *P* values were determined by a two-tailed unpaired Student’s *t* test.

We next assessed SR Ca^2+^ content using caffeine, an RyR agonist. Caffeine-induced Ca^2+^ release was markedly enhanced in VMP1-deficient cardiomyocytes relative to wild-type controls (Fig. 3, A to C), indicating increased SR Ca^2+^ content. To determine whether this increased SR Ca^2+^ content destabilizes SR Ca^2+^ handling, we performed line-scan confocal imaging of spontaneous Ca^2+^ release events. VMP1-deficient cardiomyocytes exhibited a higher proportion of cells displaying spontaneous Ca^2+^ waves, with increased wave frequency and propagation velocity (Fig. 3, D and E). In addition, the Ca^2+^ spark frequency was elevated (Fig. 3, F and G), consistent with enhanced spontaneous RyR2 openings resulting from increased SR Ca^2+^ content. The concomitant increase in sparks and propagating waves represents hallmark features of arrhythmogenic Ca^2+^ instability, providing mechanistic insight into how VMP1 deficiency predisposes cardiomyocytes to triggered activity.

To further explore the effects of VMP1 deficiency, we assessed the excitability of ventricular cardiomyocytes by recording AP in isolated primary cardiomyocytes using patch-clamp techniques (Fig. 3, A and H). During AP generation, SR Ca^2+^ content influences intracellular Ca^2+^ homeostasis and modulates AP morphology, duration, and ECC. We quantified several parameters, including AP duration at 10, 30, 50, 70, and 90% repolarization (APD10, APD30, APD50, APD70, and APD90, respectively); peak amplitude; rise time; and maximum rise slope. Compared to wild-type cardiomyocytes, VMP1-deficient ventricular cardiomyocytes exhibited prolonged APD90 (Fig. 3, H and I), along with increased rise time and decreased maximal rise slope (Fig. 3, J and K). The rise time and maximal rise slope are parameters that were used to evaluate depolarization of AP, which indicated delayed depolarization. We further examined the Na_v_1.5 channel function (the principal Na^+^ channel responsible for the rapid depolarization phase of the cardiac AP) in a heterologous expression system. Patch-clamp recordings revealed a marked reduction in Na_v_1.5 current density in knockout (KO) cells (fig. S5, A and B), indicating impaired channel function. These findings suggest that reduced Na_v_1.5 activity may contribute to the slowed depolarization kinetics in VMP1-deficient cells, although this mechanism alone does not explain the delayed repolarization. Other parameters, including APD10, APD30, APD50, APD70, and peak amplitude, remained comparable between wild-type and VMP1-deficient ventricular cardiomyocytes (Fig. 3, L to P).

Fig. 3, H to K Collectively, these results demonstrate that VMP1 deficiency leads to increased SR Ca^2+^ content, arrhythmogenic spontaneous Ca^2+^ release, and altered AP dynamics, impairing cardiomyocyte excitability.

### VMP1 forms a Ca^2+^ permeable channel

The above observations suggest that VMP1 may function as an SR Ca^2+^ release channel. To test this hypothesis, we performed single-channel electrophysiology assays using purified recombinant VMP1 with an N-terminal Flag tag (fig. S6A). On denaturing gels, VMP1 displayed a molecular weight of 45 kDa (fig. S6, B and C). However, native gel analysis revealed a notably larger size than its monomeric form, suggesting the formation of oligomers (fig. S6D). Further investigations confirmed that VMP1 undergoes self-association (fig. S6E). These findings indicate that purified VMP1 primarily exists as oligomers, a characteristic feature of ion channels.

**Fig. 4.**
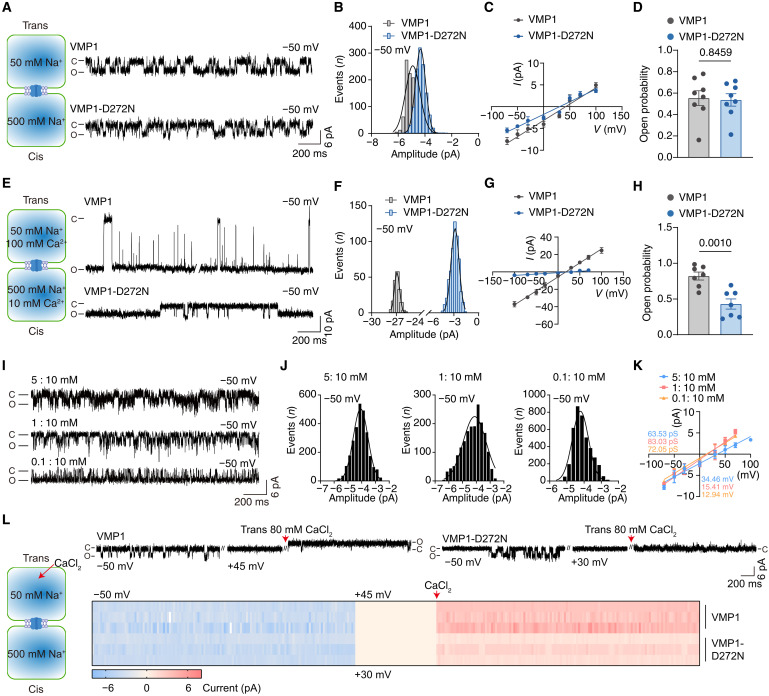
VMP1 forms a Ca^2+^-permeable channel. (**A**) Single-channel current recordings of VMP1 and VMP1-D272N at the indicated potentials in a 500 mM:50 mM NaCl solution. Left: Schematic of the recording solutions on both sides. Right: Representative single-channel currents of VMP1 and VMP1-D272N. C denotes the closed state, while O denotes the open state. (**B**) Current histogram of the traces in (A). Curves were fitted by Gaussian fit. (**C**) *I*-*V* curves of VMP1 and VMP1-D272N in a 500 mM:50 mM NaCl solution (*n* ≥ 3). (**D**) Open probability of VMP1 and VMP1-D272N in a 500 mM:50 mM NaCl solution (−50 mV). (**E**) Single-channel current recordings of VMP1 and VMP1-D272N at the indicated potentials in a Na^+^/Ca^2+^ solution. Left: Schematic of the recording solutions in the cis and trans sides. Right: Representative single-channel currents of VMP1 and VMP1-D272N. (**F**) Current histogram of the traces in (E). Curves were fitted by Gaussian fit. (**G**) *I*-*V* curves of VMP1 and VMP1-D272N in a Na^+^/Ca^2+^ solution (*n* ≥ 3). (**H**) Open probability of VMP1 and VMP1-D272N in a Na^+^/Ca^2+^ solution (−50 mV). (**I**) Single-channel current recordings of VMP1 in a mixed Na^+^/Ca^2+^ solution with different Ca^2+^ gradients. (**J**) Current histogram of the traces in (I). Curves were fitted by Gaussian fit. (**K**) *I*-*V* curves of VMP1 in a 500 mM:50 mM NaCl solution with different ratios of calcium (*n* ≥ 3). (**L**) Ca^2+^ titration of VMP1 and VMP1-D272N in a 500 mM:50 mM NaCl solution. Up: Representative single-channel currents of VMP1 and VMP1-D272N. Down: Heatmap of current signals from the traces. Each row represents an individual case. Data represent the means ± SEM. *n* indicates the number of independent experiments. Exact *P* values were determined by a two-tailed unpaired Student’s *t* test.

To evaluate VMP1 channel activity, purified VMP1 protein was added to asymmetric sodium chloride (NaCl) solutions (50:500 mM NaCl), with the Flag peptide serving as a negative control. VMP1 induced classic single-channel currents (Fig. 4A), whereas the control Flag peptide did not (fig. S7A). To further ensure that these events did not arise from nonspecific membrane perturbation, we incorporated a previously characterized viral ion channel (*23*), MERS-E, and generated a transmembrane domain–deleted mutant (MERS-E-ΔTM) as a membrane-protein negative control. No single-channel currents were detected from MERS-E-ΔTM (fig. S7A), thereby excluding potential artifact channel-like activities arising from nonspecific membrane perturbation.

Conductance and open probability analysis revealed that VMP1 exhibited a single-channel conductance of ∼74.84 pS and an open probability of 55.3%, supporting its function as an ion channel. Current-voltage relation (*I*-*V*) curve analysis in asymmetric NaCl solutions revealed a linear relationship with a reversal potential of ∼+42.25 mV (Fig. 4, B to D), which is close to the equilibrium potential for monovalent cations (Na^+^). The permeability of Cl^−^ was also tested using asymmetric choline chloride solutions, but no typical single-channel currents were observed (fig. S7B). Together, these findings indicate that VMP1 forms a cation-selective channel (*E*_rev_ = +42.25 mV; *P*_Na+_/*P*_Cl−_ = 10.52).

To assess the Ca^2+^ permeability of the VMP1 channel, we conducted two experiments. First, we evaluated VMP1’s channel activity in Na^+^/Ca^2+^ mixed solutions (Fig. 4, E to K). Second, we performed Ca^2+^ titration to isolate Ca^2+^ currents (Fig. 4, L and M). In Na^+^/Ca^2+^ mixed solutions, VMP1 generated stepwise currents (Fig. 4E). The *I*-*V* relationship showed that adding 10:100 mM (cis:trans) Ca^2+^ shifted the reversal potential of an asymmetric 500:50 mM Na^+^ solution from +42.25 to +17.97 mV and increased the channel conductance to 321.3 pS (Fig. 4, E to H), indicating high Ca^2+^ permeability (*E*_rev_ = +17.97 mV; *P*_Ca2+_*/P*_Na+_ = 0.763). To evaluate VMP1’s channel activity under Ca^2+^ conditions more comparable to physiological ranges, we further tested lower Ca^2+^ gradients by introducing 5:10 mM, 1:10 mM, and 0.1:10 mM calcium chloride (CaCl_2_) (cis:trans) into the asymmetric 500:50 mM Na^+^ solution. These conditions produced reversal potentials of +34.46, +15.41, and +12.94 mV, respectively, with corresponding single-channel conductance values of 63 to 83 pS, like those observed in NaCl alone (74.8 pS). The progressive left-shift in reversal potential across decreasing Ca^2+^ gradients confirms that VMP1 remains Ca^2+^-permeable over a wide range of luminal Ca^2+^ concentrations (Fig. 4, I to K). In Ca^2+^ titration experiments, VMP1 was added to a 500:50 mM (cis:trans) Na^+^ solution. Stable inward Na^+^ currents confirmed channel formation (Fig. 4L). To isolate Ca^2+^ currents, we adjusted the membrane potential to the reversal potential (+45 mV) to eliminate Na^+^ currents. Subsequent Ca^2+^ titration revealed stepwise outward currents at 80 mM Ca^2+^ (Fig. 4, L and M, and fig. S7C), supporting VMP1’s Ca^2+^ permeability.

Previous studies identified D272 as a critical residue of VMP1 for ER Ca^2+^ release (*18*). To substantiate this at the single-channel level, we purified the D272N mutant of VMP1 for electrophysiological analysis. The D272N mutant retained channel activity in asymmetric Na^+^ solutions, with electrophysiological properties similar to the wild-type VMP1 (*E*_rev_ = 30.69 mV; *P*_Na+_/*P*_Cl−_ = 4.78) (Fig. 4, A to D). However, in Na^+^/Ca^2+^ mixed solutions, the D272N mutant displayed a markedly reduced conductance (31.27 pS) and significantly decreased open probability compared to wild-type VMP1 (Fig. 4, E to H). Similarly, in Ca^2+^ titration experiments, the D272N mutant exhibited minimal channel currents upon the addition of 80 mM Ca^2+^ (Fig. 4, L and M, and fig. S7C). Collectively, these findings demonstrate that VMP1 forms a Ca^2+^-permeable ion channel.

### D272 of VMP1 is essential for cardiac function

Given that D272 of VMP1 is critical for sensing Ca^2+^ in single-channel electrophysiology assays, we investigated its in vivo importance in the heart using previously reported D272N point mutation knockin mice (*18*). In *Myh6^Cre^Vmp1^fl/D272N^* mice, the floxed *Vmp1* allele was deleted by *Myh6^Cre^*, while the other *Vmp1^D272N/+^* allele, a germline mutation, likely abolished VMP1’s ability to release SR Ca^2+^. Consequently, cardiomyocytes in *Myh6^Cre^Vmp1^fl/D272N^* mice expressed only the VMP1 D272N variant, whereas both wild-type VMP1 and D272N VMP1 were expressed in other tissues.

Similar to *Myh6^Cre^Vmp1^fl/fl^* mice (Fig. 1E), all *Myh6^Cre^Vmp1^fl/D272N^* mice died between postnatal days 12 and 14 (fig. S8A). Histological analysis revealed dilated cardiomyopathy in these mice (fig. S8, B and C), and severe cardiac arrhythmias were observed as early as day 11 (fig. S8D).

**Fig. 5.**
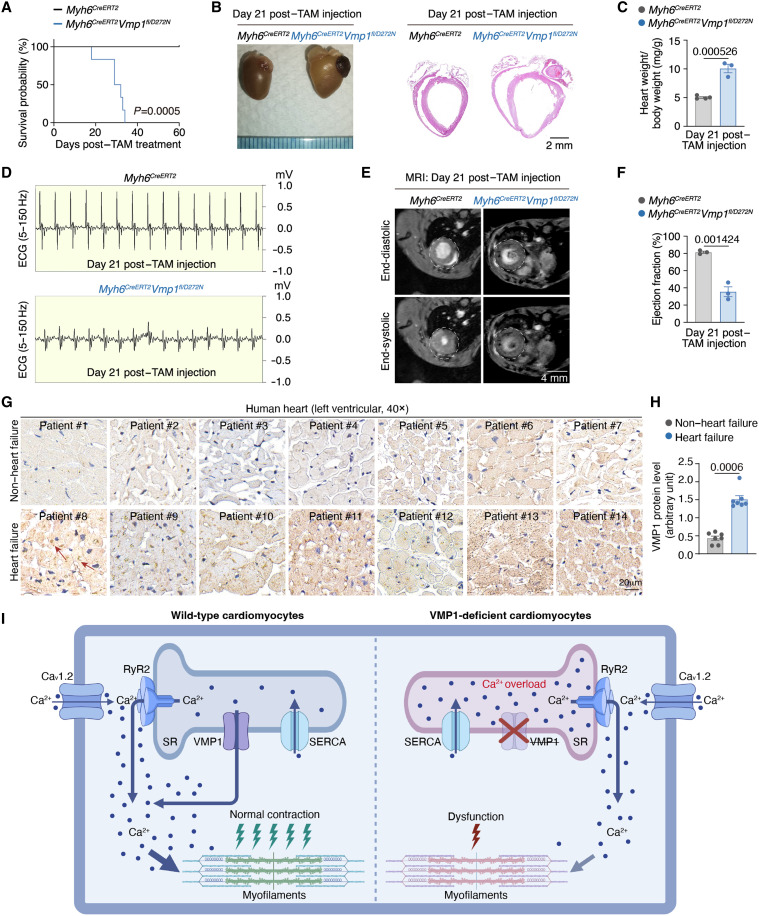
D272 of VMP1 is essential for cardiac function in vivo and up-regulation of VMP1 in patients with heart failure. (**A**) Survival curve of control and *Myh6^CreERT2^Vmp1^fl/D272N^* mice following tamoxifen injection (*n* = 6 mice per group). (**B**) Representative images of hearts and H&E staining of longitudinal heart sections from control and *Myh6^CreERT2^Vmp1^fl/D272N^* mice at 21 days post–tamoxifen injection. (**C**) Heart weight/body weight ratio in control mice (*n* = 4) and *Myh6^CreERT2^Vmp1^fl/D272N^* mice (*n* = 3) at 21 days post–tamoxifen injection. The control group data are shared with Fig. 1J. (**D**) Representative ECG recordings from control and *Myh6^CreERT2^Vmp1^fl/D272N^* mice at 21 days post–tamoxifen injection. (**E**) Representative MRI images of the short axis of the heart in control and *Myh6^CreERT2^Vmp1^fl/D272N^* mice at 21 days post–tamoxifen injection. The heart is outlined with a white dashed line. (**F**) Percentage of ejection fraction measured by MRI (*n* = 3 mice per group). (**G**) Representative immunohistochemistry images of human left ventricle tissues stained with an anti-VMP1 antibody, captured at 40× magnification. (**H**) Quantification of VMP1 expression levels by ImageJ. (**I**) Working model of VMP1 functioning as an SR Ca^2+^ release channel to maintain cardiomyocyte contraction. Data represent the means ± SEM. *n* indicates the number of mice. Exact *P* values were determined by a log-rank (Mantel-Cox) test in (A) and a two-tailed unpaired Student’s *t* test in (C), (F), and (H).

Consistently, all *Myh6^CreERT2^Vmp1^fl/D272N^* mice succumbed to sudden death within 5 weeks following tamoxifen treatment (Fig. 5A). Histological examination confirmed hallmark features of dilated cardiomyopathy (Fig. 5, B and C), and severe arrhythmias were observed in *Myh6^CreERT2^Vmp1^fl/D272N^* mice 21 days post–tamoxifen treatment (Fig. 5D). Consistent with the severe arrhythmias, MRI analysis revealed an increased end-systolic internal volume of the left ventricle (Fig. 5E), leading to a significant reduction in ejection fraction in *Myh6^CreERT2^Vmp1^fl/D272N^* mice (Fig. 5F).

Cardiomyocytes isolated from *Myh6^CreERT2^Vmp1^fl/D272N^* mice exhibited a higher SR Ca^2+^ content (fig. S8E), increased spontaneous Ca^2+^ waves, and elevated Ca^2+^ wave/spark frequencies compared to control cardiomyocytes (fig. S8, F to I), mirroring the SR Ca^2+^ handling defects of VMP1-deficient cardiomyocytes. Together, these findings demonstrate that D272, an amino acid required for VMP1 to sense SR Ca^2+^, is essential for maintaining normal heart function in mice.

### Increased VMP1 expression in patients with heart failure

It has been shown that VMP1 expression in the heart is elevated during cardiac hypertrophy in mice (*24*), suggesting a potential role for VMP1 in heart failure. To investigate its involvement in human heart disease, we analyzed VMP1 expression in patients with heart failure. Left ventricular samples were obtained from medicolegal autopsies, including seven individuals with heart failure (patient nos. 8 to 12 with hypertrophic cardiomyopathy and patient nos. 13 and 14 with dilated cardiomyopathy) and seven (patient nos. 1 to 7) without heart failure. Immunohistochemical staining revealed significantly elevated VMP1 proteins in cardiomyocytes from individuals with heart failure compared to those without (Fig. 5, G and H).

We further assessed a mouse model of heart failure and found that mice with induced heart failure exhibited increased VMP1 expression in cardiomyocytes compared to controls (fig. S8J), consistent with human heart failure samples. These findings suggest that increased VMP1 expression in failing hearts may contribute to heart failure pathogenesis or reflect a compensatory response to disease progression.

## DISCUSSION

Since the identification by ryanodine in 1985 (*25*), the ryanodine receptor has been recognized as the principal, if not sole, SR Ca^2+^ release channel driving muscle ECC. After 40 years, we unexpectedly identified VMP1 as a Ca^2+^ channel essential for SR Ca^2+^ release and cardiac muscle contraction. Notably, VMP1 exhibits distinct temporal expression and functional requirements in the heart. While RyR2 expression begins during embryonic development (*5*), VMP1 is minimally expressed in the fetal heart but undergoes rapid up-regulation within 24 hours after birth, reflecting a developmental shift in SR Ca^2+^ handling. Consistent with this, constitutive deletion of RyR2 in cardiomyocytes results in embryonic lethality in mice (*26*), whereas VMP1-deficient mice survive until postnatal day 12 before experiencing sudden cardiac death. This indicates that VMP1 is dispensable for fetal heart function but becomes indispensable during early neonatal development, likely aligning with increased physiological demands as neonatal mice become active around postnatal day 12. In adult mice, inducible deletion of either RyR2 (*22*) or VMP1 results in rapid heart failure, underscoring the notion that both these two SR Ca^2+^ release channels are essential for maintaining cardiac function in adulthood. These findings redefine the molecular mechanisms underlying cardiac ECC by identifying VMP1 as a temporally distinct and complementary SR Ca^2+^ release channel that operates alongside RyR2 in the postnatal heart (Fig. 5I).

Mechanistically, we show that VMP1 deficiency leads to increased SR Ca^2+^ content and dysregulated APs in cardiomyocytes. Single-channel electrophysiology reveals that VMP1’s Ca^2+^ channel activity is Ca^2+^ regulated, with higher activity at elevated Ca^2+^ concentrations. This Ca^2+^ regulation is mediated by the luminal residue D272, positioning VMP1 as a monitor of luminal Ca^2+^ levels in the SR. This mechanism is distinct from RyR2 in that VMP1 is regulated solely by luminal Ca^2+^, whereas RyR2 is known to be sensitive to both cytosolic and luminal Ca^2+^ (*5*). Thus, VMP1 provides an additional luminal Ca^2+^-dependent regulatory component that helps fine-tune SR Ca^2+^ release. Although we cannot fully exclude subtle alterations in RyR2 regulation, the major defects observed in VMP1-deficient cardiomyocytes are most consistent with the loss of VMP1’s luminal Ca^2+^ sensing. Together, these complementary mechanisms contribute to the precision of ECC and normal heartbeat.

While VMP1 has previously been implicated in autophagy and SERCA regulation (*12*, *13*), our findings suggest that its Ca^2+^ channel function may be the primary determinant of its cardiac role. ATG5 (autophagy related 5)–deficient mice, which lack autophagy, exhibit only mild cardiac phenotypes at late ages without arrhythmias or sudden cardiac death (*27*), suggesting that autophagy defect alone cannot explain the lethal phenotype observed in VMP1-deficient hearts. Similarly, the elevated SR Ca^2+^ levels in VMP1-deficient cardiomyocytes argue against a positive regulatory role for SERCA (*13*). The absence of VMP1 did not affect the expression levels of RyR2 or SERCA2 (fig. S8K) in cardiomyocytes nor their localization in SR (fig. S4), indicating that VMP1 unlikely controls SR levels indirectly through RyR2 or SERCA2. In contrast, the ER/SR luminal Ca^2+^-buffering chaperone calreticulin was up-regulated in both VMP1-KO and VMP1-D272N cardiomyocytes (fig. S8K). Given that calreticulin modulates SR Ca^2+^ storage capacity and VMP1 disruption increases SR Ca^2+^ content, this calreticulin up-regulation likely represents an adaptive response to increased SR Ca^2+^ content. Na_v_1.5 activity was reduced in the absence of VMP1, suggesting a complex role for VMP1 in cardiac ion handling. The contribution of altered Na_v_1.5 function in VMP1-deficient hearts warrants further investigation. VMP1 also functions as a lipid scramblase that regulates cellular metabolism (*14*–*16*), which may influence membrane permeability, potentially leading to Ca^2+^ leakage and contributing to the cardiac phenotype observed in the absence of VMP1. Collectively, while we propose that VMP1’s Ca^2+^ channel activity plays an important role in the heart, its other functions, including roles in autophagy, SERCA regulation, Na_v_1.5 modulation, and lipid scramblase activity, may also contribute to the phenotypes observed in this study.

Notably, mice carrying the D272N mutation, which disrupts VMP1’s ability to sense Ca^2+^, fully recapitulate the phenotypes of VMP1-deficient mice. This provides strong evidence that the observed cardiac dysfunction arises specifically from the loss of VMP1’s Ca^2+^ channel activity.

Alterations in Ca^2+^ handling in cardiomyocytes are central to the pathogenesis of various heart diseases (*28*–*30*). RyR2, because of its established role in SR Ca^2+^ release and its dysregulation in heart diseases, is a major target for the development of therapies for arrhythmias and heart failure (*7*, *8*, *31*). The severe arrhythmias and heart failure observed in the absence of VMP1 suggest that genetic variants in VMP1 or its regulatory components could underlie certain forms of inherited cardiac arrhythmias or cardiomyopathies. Unlike RyR2, which mainly exhibits mutations or aberrant modifications in heart diseases (*6*, *31*), we observed significant up-regulation of VMP1 in failing human hearts. This may represent a compensatory response to maintain Ca^2+^ release under pathological conditions. These findings position VMP1 as a potential biomarker cardiac dysfunction. However, whether VMP1 up-regulation is protective or pathological in the context of heart failure remains to be determined. The identification of D272 as a critical residue for Ca^2+^ sensing opens opportunities to develop targeted strategies for modulating VMP1 activity.

Beyond its role in the heart, our findings open avenues for exploring VMP1 as a Ca^2+^ channel in other tissues and organs. Dysregulation of ER Ca^2+^ homeostasis has been implicated in a variety of diseases, ranging from neurodegenerative to metabolic disorders (*32*–*34*). Aberrant VMP1 expression has also been associated with infection (*17*), inflammation (*35*, *36*), and cancer (*37*, *38*). Given VMP1’s critical role in SR/ER Ca^2+^ release, its dysregulation may contribute to diverse disease pathogenesis, suggesting that targeting VMP1 could have far-reaching therapeutic implications beyond cardiac disease.

In conclusion, we identify VMP1 as a SR Ca^2+^ release channel essential for cardiac function. By delineating its distinct role in SR Ca^2+^ regulation, we provide insights into the mechanisms of ECC and establish VMP1 as a promising target for understanding and treating cardiac and systemic diseases linked to ER/SR Ca^2+^ dysregulation.

## MATERIALS AND METHODS

### Mice

*Vmp1^fl/fl^* and *Vmp1^D272N/+^* mice were previously described (*18*). *Myh6^Cre^* (C001041) and *Myh6^CreERT2^* (C001009) transgenic mice, originally from Cyagen Biosciences, were maintained on a C57BL/6 background. Age- and sex-matched littermates were used as controls in all experiments. All mice were housed under specific pathogen–free conditions at the Laboratory Animal Research Center of Tsinghua University (Beijing, China), which is accredited by the Beijing Administration Office of Laboratory Animals. All experimental procedures were approved by the Institutional Animal Care and Use Committee of Tsinghua University (approval number: 17-PM1.G23-1).

### Cell lines

HEK293T cells (cat. no. CRL-3216; RRID: CVCL_0063) were obtained from American Type Culture Collection and cultured in Dulbecco’s modified Eagle’s medium (Gibco) supplemented with 10% fetal bovine serum (Gemini), 2 mM l-glutamine (Macgene), and penicillin (100 units/ml) and streptomycin (100 μg/ml; Macgene). Cells were maintained at 37°C in a humidified incubator with 5% CO_2_. Mycoplasma contamination was tested using the TransDect PCR Mycoplasma Detection Kit (TRAN) and confirmed to be negative.

### Inducible deletion of VMP1 in *Myh6^CreERT2^* mice by tamoxifen treatment

Tamoxifen (Sigma-Aldrich, cat. no. T5648) was dissolved in corn oil (Sigma-Aldrich, cat. no. C8267) at a concentration of 15 mg/ml, and the solution was mixed until it became completely clear and transparent. Mice were administered 100 μl of the solution (15 mg/ml) by intraperitoneal injection every other day for a total of four injections.

### Hematoxylin and eosin (H&E) staining

Excised hearts were immediately rinsed in phosphate-buffered saline (PBS) and fixed in 4% paraformaldehyde at 4°C for at least 24 hours. After fixation, the samples were dehydrated through a graded ethanol series, embedded in paraffin, and sectioned at a thickness of 5 μm. The sections were stained with hematoxylin and eosin (H&E) to visualize tissue architecture.

### ECG recording

Mice were lightly anesthetized with 0.5 to 1% isoflurane in a 95% oxygen mix to maintain light sedation. Each mouse was placed on a warming pad, and subcutaneous needle electrodes were inserted into the right forelimb and hindlimbs. Continuous ECG recordings were obtained for at least 5 min, starting once the heart rate had stabilized. The procedure was performed within a metal shielding cage to minimize electrical interference. Biopac Student Lab software (BIOPAC) was used for ECG collection and data analysis.

### Cardiac function assessment by MRI

Cardiac function in mice was evaluated using a 9.4-T small animal magnetic MRI system (Bruker, BioSpec 94/30 USR). Mice were anesthetized with 1.5 to 2% isoflurane in oxygen, and their heart rate was continuously monitored using a needle electrode–based ECG system. Imaging was initiated once the heart rate had stabilized. The ejection fraction was calculated using the following formula: the area of the left ventricle at end-diastole minus the area at end-systole, divided by the area at end-diastole, and then multiplied by 100.

### Echocardiographic analysis

To evaluate the cardiac function, mice were lightly anesthetized with 0.5 to 1% isoflurane in a 95% oxygen mix to ensure minimal sedation. Each mouse was placed on a warming pad to maintain body temperature. Echocardiography was performed using the VINNO-6 ultrasound system. Comprehensive imaging and measurements were obtained from the left ventricle in long-axis, short-axis, and apical four-chamber views.

### Ventricular myocytes isolation

Mice were administered 0.5% heparin sodium (Beijing Vokai Biotechnology Company, China) via intraperitoneal injection. The heart was removed from the chest and connected to the perfusion system. Initially, the heart was perfused with Ca^2+^-free Tyrode’s solution for 5 min at 36.5°C, followed by Tyrode’s solution containing collagenase type II (Worthington Biochemical Corporation, US), collagenase type IV (Worthington Biochemical Corporation, US), and collagenase NB 8 (SERVA Electrophoresis GmbH, Germany) for 15 to 20 min. The ventricular tissue was dissected and torn into small pieces, and a single myocyte suspension was obtained through repeated pipetting and filtration using a 200-mesh filter. The isolated ventricular myocytes were then recalcified (0 to 1.8 mM Ca^2+^) to restore the physiological function and subsequently placed on ice for further experiments.

### Live-cell Ca^2+^ imaging

Isolated ventricular myocytes were seeded onto poly-d-lysine–coated coverslips for 1 hour and then incubated with 4 μM Fura-2 AM (Invitrogen, US) for 60 min at 37°C. After incubation, the cells were washed three times and maintained in Ca^2+^-free buffer for 30 min before the experiment. Ca^2+^ imaging was conducted using an inverted Nikon Ti-E microscope (Nikon Instruments Inc., US), and changes in intracellular Ca^2+^ concentration were measured using the Fura-2 fluorescence ratio (F340/F380). Time-lapse images were captured to determine the ratio values of ventricular myocytes before and after stimulation with caffeine (2 mg/ml). Cells exhibiting changes in the Fura-2 ratio smaller than 20% were excluded from the statistical analysis. Ca^2+^-free Hanks’ balanced salt solution buffer was used throughout the imaging process to prevent interference from calcium channels in the plasma membrane.

### Calcium spark and calcium wave measurement

Isolated ventricular myocytes were plated onto poly-d-lysine–coated coverslips for 1 hour and then incubated with Fluo-4 AM (Thermo Fisher Scientific, US) and probenecid for 60 min at 37°C. After incubation, the cells were washed three times and maintained in Ca^2+^-free buffer. Ca^2+^ spark and wave were measured using line-scan mode of the Leica TCS-SP8 STED system (Leica Microsystems, Germany). Analysis of *x*-*t* images for Ca^2+^ sparks was performed using the SparkMaster plug-in in ImageJ. Analysis of *x*-*t* images for Ca^2+^ waves was also performed using ImageJ software.

### Patch-clamp recording

For AP detection, whole-cell patch-clamp recordings were performed at room temperature using a Multiclamp 700B amplifier (Molecular Devices, US). Pipettes were pulled using a Sutter P-1000 puller (Sutter Instrument Company, US) with an open-tip resistance ranging from 5 to 10 MΩ. The pipette solution contained the following components: 5 mM Na_2_ATP, 130 mM K-aspartate, 10 mM Hepes, 1 mM MgCl_2_, 11 mM EGTA, and 2 mM CaCl_2_ (pH 7.2, adjusted with KOH). The bath solution contained 140 mM NaCl, 5 mM KCl, 10 mM Hepes, 1.25 mM MgCl_2_, 10 mM glucose, and 1.8 mM CaCl_2_ (pH 7.4, adjusted with NaOH). After achieving the whole-cell configuration, APs were recorded in current-clamp mode. Ventricular myocytes were stimulated with short pulses (2-ms duration, 1-Hz frequency) of 1000- to 2000-pA current. Data were analyzed using Clampfit 11.2 software (Axon Instruments, US), and AP parameters were calculated using the AP search plug-in.

For Na_v_1.5 current recordings, wild-type and VMP1-KO HEK293T cells were transiently transfected with the pcDNA5/FRT-Na_v_1.5 plasmid. Whole-cell patch-clamp recordings were performed at room temperature using a Multiclamp 700B amplifier (Molecular Devices, US). Pipettes were pulled using a Sutter P-1000 puller (Sutter Instrument Company, US) with an open-tip resistance ranging from 2 to 5 MΩ. The pipette solution contained the following components: 130 mM CsF, 10 mM NaCl, 10 mM Hepes, and 10 mM EGTA, pH 7.2, with CsOH. The bath solution contained 140 mM NaCl, 5 mM KCl, 1 mM CaCl_2_, 1.25 mM MgCl_2_, 10 mM Hepes, and 10 mM glucose, pH 7.4, with NaOH. After achieving the whole-cell configuration, Na_v_1.5 current was recorded in voltage-clamp mode. Data were analyzed using Clampfit 11.2 software (Axon Instruments, US).

### Purification of recombinant VMP1

To purify recombinant VMP1 proteins, 8 million HEK293T cells were seeded in 15-cm plates 16 hours before transfection. Transfection was performed using 20 mg of plasmids encoding FLAG-VMP1 or FLAG-VMP1-D272N and Chemifect (Fengrui, cat. no. FR-01), following the manufacturer’s instructions. After 36 hours, cells were harvested, washed twice with ice-cold PBS, and lysed in 1% *n*-dodecyl β-d-maltoside lysis buffer. The lysis buffer contained 20 mM tris-Cl (pH 8.0), 2 mM CaCl_2_, 200 mM NaCl, 1% *n*-dodecyl β-d-maltoside, 0.2% (w/v) cholesteryl hemisuccinate, 0.012% (w/v) glyco-diosgenin (GDN; Anatrace, cat. no. GDN101), and 1× EDTA-free protease inhibitor cocktail (Yeasen, cat. no. 20123ES50). The buffer volume was approximately five times the volume of the cell pellet. Cell lysis was performed for 2 hours with gentle rotation at 4°C. The lysate was centrifuged at 15,000 rpm for 30 min at 4°C, and the supernatant was incubated with anti-DYKDDDDK G1 affinity resin (GenScript, cat. no. L00907) for 3 hours with gentle rotation at 4°C. The resin was washed five times with wash buffer containing 20 mM tris-Cl (pH 8.0), 200 mM NaCl, 2 mM CaCl_2_, and 0.008% GDN. Bound proteins were eluted with 500 ml of wash buffer supplemented with FLAG peptide (0.125 mg/ml; GenScript, cat. no. RP10586) and incubated for 2 hours with gentle rotation at 4°C. The eluted protein solution was filtered through a 0.22-mm spin filter and directly loaded onto an Enrich SEC650 column (Bio-Rad) for size exclusion chromatography. Protein-containing fractions were collected and analyzed (*39*).

### Single-channel electrophysiological recording

Purified VMP1 or VMP1-D272N protein was incorporated into lipid bilayers to assess their functionality. The bilayer lipids consisted of phosphatidylcholine and phosphatidylserine at a 3:2 ratio (Avanti Polar Lipids, US). The detection system featured cis and trans compartments, with the cis side containing a solution of 500 mM NaCl and 5 mM Hepes (pH 6.35) and the trans side containing 50 mM NaCl and 5 mM Hepes (pH 6.35). Proteins were introduced to the cis side, and their incorporation into the lipid membrane was facilitated by the electrochemical gradient and agitation.

Membrane currents were recorded in voltage-clamp mode using a bilayer clamp amplifier (BC-535, Warner Instruments, US) and filtered at 1 to 2 kHz. The signals were digitized with pClamp 10.2 software (Molecular Devices, US). Data were fitted to Gaussian functions or single/biexponential equations to calculate single-channel conductance and open times. Signals with opening times shorter than 0.5 to 1.5 ms were excluded from analysis. The Nernst equation or Goldman-Hodgkin-Katz flux equation was applied to determine equilibrium potentials in single-ion or multi-ion solutions.

### Western blot

Cell lysates from tissues, organs, and isolated adult cardiomyocytes were prepared using Cell Lysis Buffer for Western blotting and immunoprecipitation (Beyotime, cat. no. P0013), supplemented with an EDTA-free protease inhibitor cocktail. For HEK293T cells, after the indicated treatments, cells were collected, washed with cold PBS, and lysed on ice for 15 min in lysis buffer containing 1% Triton X-100, 40 mM Hepes (pH 7.4), 10 mM β-glycerophosphate, and 10 mM pyrophosphate, supplemented with EDTA-free protease inhibitor cocktail.

The soluble fractions of the cell lysates were obtained by centrifugation at 15,000 rpm for 10 min at 4°C. Proteins were denatured by adding 6× SDS sample buffer and boiling the samples at 95°C for 10 min. The samples were subjected to SDS–polyacrylamide gel electrophoresis (LABLEAD, cat. no. P41215), native gel electrophoresis, and subsequent immunoblotting. The following antibodies were used: rabbit monoclonal anti–TMEM49/VMP1 (D1Y3E) (Cell Signaling Technology, cat. no. 12929), WDR24 (Proteintech, cat. no. 20778-1-AP), rabbit monoclonal anti-HA-Tag (C29F4) (Cell Signaling Technology, cat. no. 3724), and mouse monoclonal anti-FLAG (DYKDDDDK) (Sigma-Aldrich, cat. no. F1804).

### Immunocytochemistry

Heart tissue sections were prepared by baking paraffin-embedded slides at 60°C for 1 hour, followed by deparaffinization and rehydration. Antigen retrieval was performed using heat-induced retrieval: Slides were immersed in citrate buffer, heated to boiling for 2 min, and then cooled to room temperature. Afterward, the slides were washed five times with PBS, 3 min per wash.

To permeabilize and block endogenous peroxidase activity, the sections were treated with 3% hydrogen peroxide in methanol for 10 min in the dark. Nonspecific binding was blocked by applying 70 ml of 10% normal goat serum to each slide, incubating them at room temperature for 20 min in a humidified chamber.

The slides were then incubated overnight at 4°C with a rabbit anti-human VMP1 polyclonal antibody (1:50, CUSABIO, cat. no. CSB-PA856940LA01HU). After equilibration to room temperature for 30 min, the slides were washed five times with PBS with Tween 20 (PBST), 3 min per wash. A goat anti-rabbit horseradish peroxidase–conjugated secondary antibody (Zhongshanjin Qiao, cat. no. PV-6000) was applied, and the slides were incubated at room temperature for 30 min. This was followed by five additional PBST washes (5 min each).

After washes with PBST, the sections were developed using 3,3′-diaminobenzidine (Zhongshan Jinqiao, cat. no. ZLI-9018). The color development was monitored under a microscope, and the reaction was terminated by immersing the slides in tap water.

Last, the sections were counterstained with hematoxylin for nuclear visualization, mounted with neutral balsam, and observed under a microscope. Images were captured at appropriate fields of view for analysis.

We quantified VMP1 expression in human left ventricle sections by first acquiring whole-slide scans at 40× magnification. The 3,3′-diaminobenzidine–positive (brown) area was then measured in ImageJ by applying a consistent optical density threshold. VMP1 expression levels were quantified as the percentage of positively stained area relative to the total tissue area. All analyses were performed blindly, and statistical comparisons between groups were made using a two-tailed unpaired Student’s *t* test.

### Human heart samples

In collaboration with the Forensic Medicine School of Shanxi Medical University, tissue samples were obtained from deceased individuals with a history of heart failure, as well as from those without heart failure. Immunohistochemical analysis was performed to assess the expression of VMP1 in these samples. The study was approved by the Ethics Committee of Shanxi Medical University and conducted in accordance with the Declaration of Helsinki. The use of human heart samples was authorized by the Institutional Review Board of Shanxi Medical University (license number 2024048). All experiments were carried out in compliance with relevant ethical guidelines.

### Isoproterenol-induced heart failure model in mice

Seven-week-old C57BL/6 mice (average body weight, 28.8 ± 0.6 g) were randomly assigned to two groups: a control group (*n* = 3) and an isoproterenol (ISO)–induced heart failure group (*n* = 3). Control mice received daily intraperitoneal injections of saline. Heart failure was induced in the ISO group by daily intraperitoneal administration of ISO (Merck, HY-B0468) dissolved in physiological saline (0.9% NaCl) at a dose of 60 mg kg^−1^ day^−1^. All mice were euthanized 21 days after the first ISO injection. Hearts were then perfused, and left ventricular tissues were collected for protein extraction. Western blot analysis was performed to compare VMP1 expression levels between the two groups.

### Statistical analysis

Data collection and analysis were performed without blinding to experimental conditions. Statistical analysis was conducted using GraphPad Prism 8.0. Data are presented as individual values or means ± SEM, as indicated in the figure legends. *P* values and the number of replicates (*n*) are shown in the figures or figure legends. A two-tailed unpaired Student’s *t* test was used to evaluate differences between two groups. *P* < 0.05 was considered significant. All experiments were repeated independently at least twice with similar results. Representative immunoblots and micrographs were selected from biological replicates.
