# Supplementary material for: VMP1 forms a Ca2+ release channel essential for postnatal heartbeat
Source: Sci Adv. 2026 Jun 12;12(24):eadz0706. doi: 10.1126/sciadv.adz0706 (PMC13262637; doi:10.1126/sciadv.adz0706)
Supplement: Supplementary file 1 — Figs. S1 to S8 [file sciadv.adz0706_sm.pdf]

Science Advances
AAAS

Supplementary Materials for

VMP1 forms a Ca^{2+} release channel essential for postnatal heartbeat

Yuying Ma *et al.*

Corresponding author: Min Peng, pengmin@tsinghua.edu.cn; Bingqing Xia, xiabingqing@simm.ac.cn; Zhaobing Gao, zbgao@simm.ac.cn; Zhifang Wu, wuzhifang01@163.com

Sci. Adv. **12**, eadz0706 (2026)
DOI: 10.1126/sciadv.adz0706

This PDF file includes:

Figs. S1 to S8

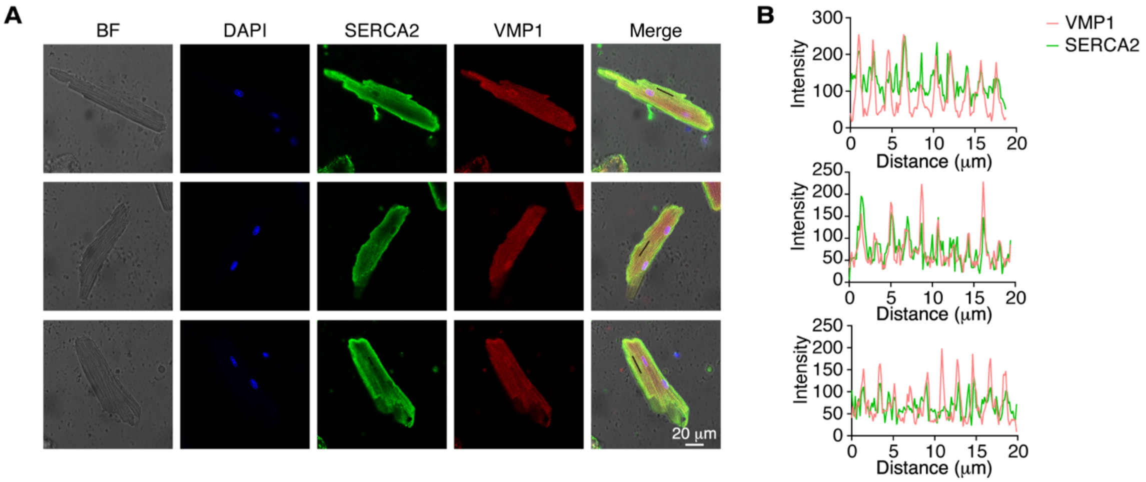

Fig. S1. Subcellular localization of VMP1 in cardiomyocytes. (**A**) Representative immunofluorescence images of isolated wild-type mouse cardiomyocytes stained for VMP1 (red) and the SR marker SERCA2a (green). Nuclei are counterstained with DAPI (blue). (**B**) Fluorescence intensity profiles corresponding to the black lines indicated in panel (A).

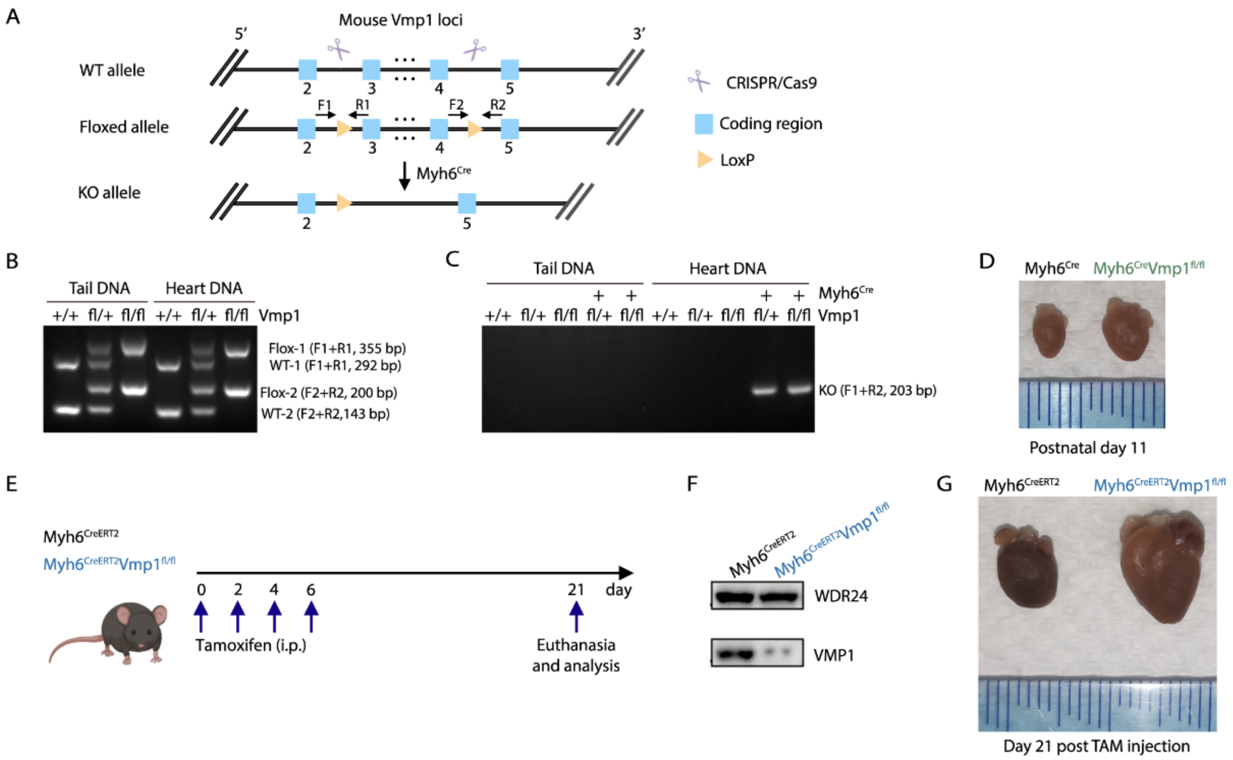

Fig. S2. Generation and characterization of cardiomyocyte-specific VMP1-deficient mice. (**A**) Gene targeting strategy illustrating the floxed *Vmp1* allele and *Myh6*^{Cre}- or *Myh6*^{CreERT2}-mediated knockout. (**B**) Genotyping of DNA extracted from mouse tail and heart tissues to identify floxed *Vmp1* alleles. (**C**) Genotyping of DNA extracted from mouse tail and heart tissues to confirm *Myh6*^{Cre}-mediated *Vmp1* knockout. (**D**) Representative images of hearts from control and *Myh6*^{Cre}*Vmp1*$^{fl/fl}$ mice at postnatal day 11. (**E**) Experimental design for tamoxifen-induced *Vmp1* deletion in *Myh6*^{CreERT2}*Vmp1*$^{fl/fl}$ mice. (**F**) Immunoblot analysis of VMP1 in isolated cardiomyocytes from control and *Myh6*^{CreERT2}*Vmp1*$^{fl/fl}$ mice at 21 days post-tamoxifen injection. Representative results from two independent experiments are shown. (**G**) Representative images of hearts from control and *Myh6*^{CreERT2}*Vmp1*$^{fl/fl}$ mice at 21 days post-tamoxifen injection.

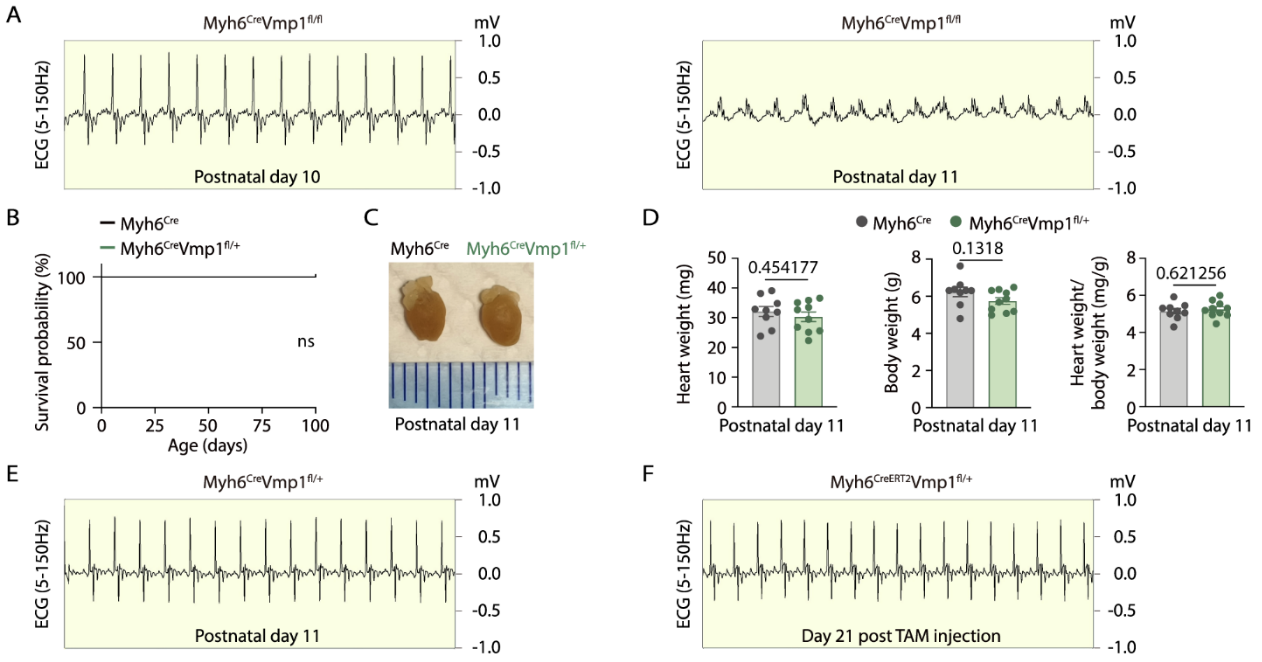

Fig. S3. Characterization of *Myh6^{Cre}Vmp1$^{fl/+}$* mice. (**A**) Representative ECG recordings from control and *Myh6^{Cre}Vmp1$^{fl/fl}$* mice at postnatal day 10 (left) and day 11 (right). (**B**) Survival curve of control and *Myh6^{Cre}Vmp1$^{fl/+}$* mice (n = 40 mice per group). (**C**) Representative images of hearts from control and *Myh6^{Cre}Vmp1$^{fl/+}$* mice at postnatal day 11. (**D**) Heart weight, body weight, and heart weight/body weight ratio in control (n = 9) and *Myh6^{Cre}Vmp1$^{fl/+}$* mice (n = 10) at postnatal day 11. The control group data are shared with Fig. 1G. (**E**) Representative ECG recording of *Myh6^{Cre}Vmp1$^{fl/+}$* mice at postnatal day 11. (**F**) Representative ECG recording of *Myh6^{CreERT2}Vmp1$^{fl/+}$* mice at 21 days post-tamoxifen injection. Data represent the mean ± s.e.m. n indicates the number of mice. Exact *P* values were determined by log-rank (Mantel-Cox) test in (B), and two-tailed unpaired Student's t-test in (D).

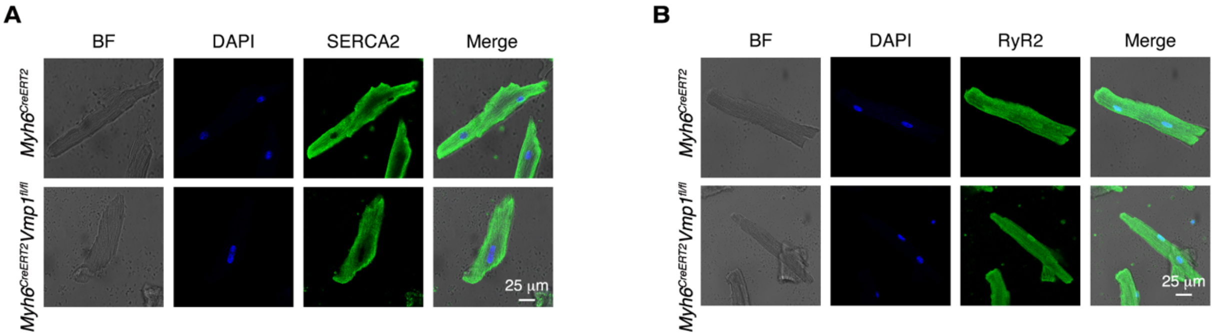

Fig. S4. VMP1 deficiency does not alter SR structure in cardiomyocytes. (**A-B**) Confocal immunofluorescence analysis of SR structure in isolated cardiomyocytes from control (*Myh6*^{CreERT2}, upper panels) and VMP-1 knockout (*Myh6*^{CreERT2}*Vmp1*$^{fl/fl}$, lower panels) mice. The SR was visualized using antibodies against SERCA2 (green) (A) and RyR2 (green) (B), with nuclei labeled by DAPI (blue).

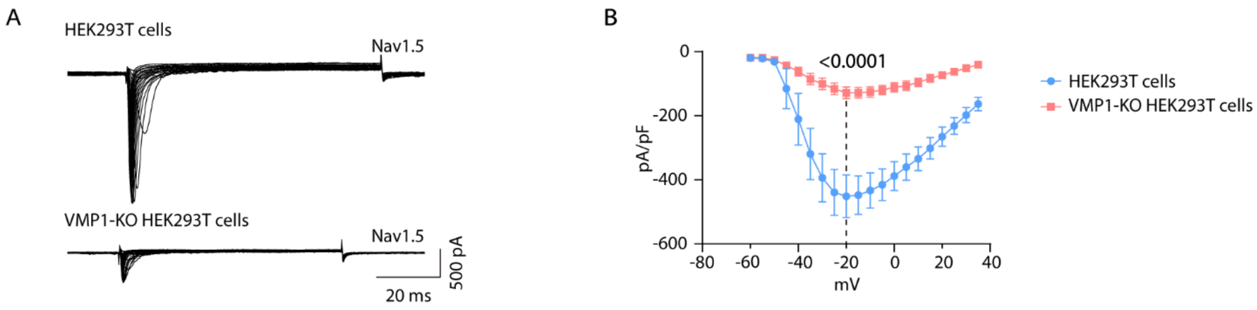

Fig. S5. **Current density of voltage-gated Na$^+$ channels is reduced in VMP1-KO HEK293T cells.** (**A**) Representative whole-cell voltage-gated sodium currents (I_{Na}) recorded from wild-type (WT) and VMP1-knockout (KO) HEK293T cells transiently transfected with a human Nav1.5 (SCN5A) expression plasmid. Scale bars: 500 pA (vertical) and 20 ms (horizontal). (**B**) I_{Na} density plot of NaV1.5-transfected WT and VMP1-KO HEK293T cells (n = 10). Current density at −20 mV was quantified. Data represent the mean ± s.e.m. Results from 3 independent experiments. Exact *P* values were determined using a two-tailed unpaired Student's *t*-test (B).

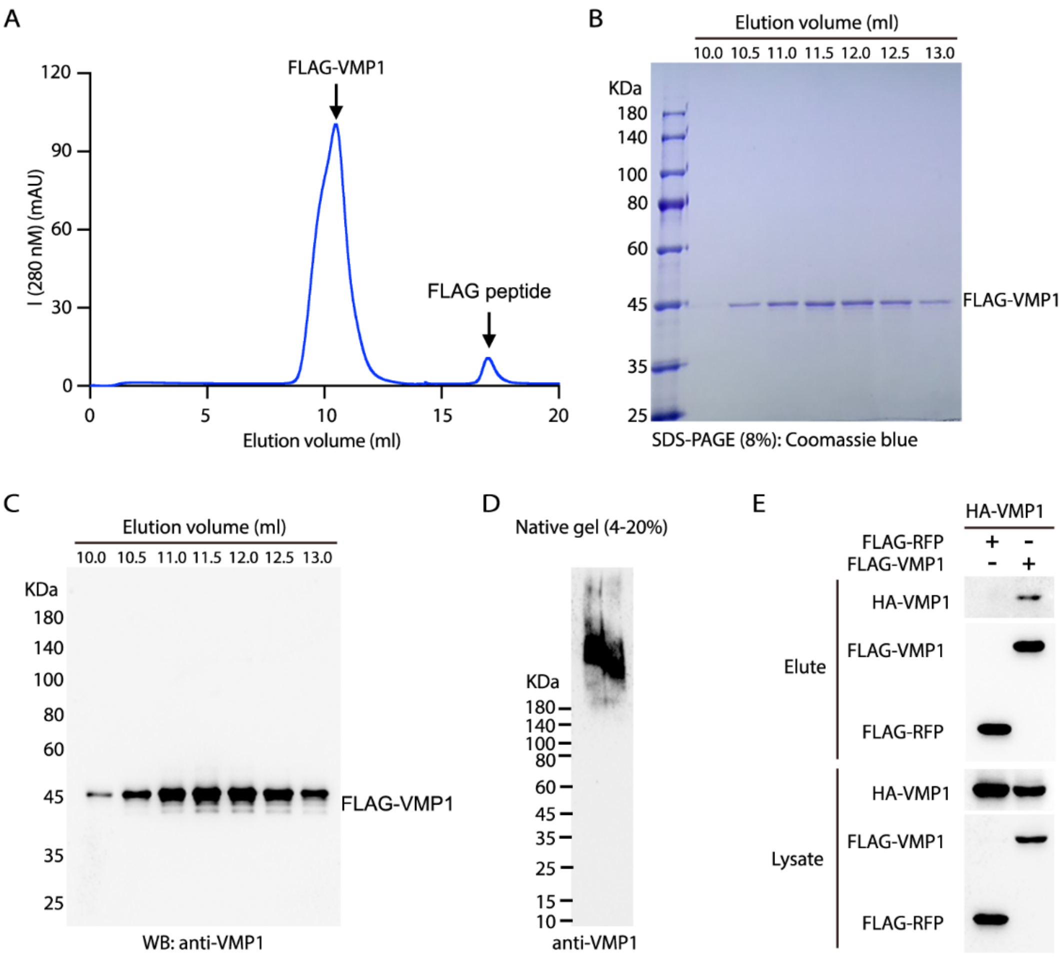

Fig. S6. **Purification and characterization of recombinant VMP1**. (**A**) Representative size exclusion chromatography (SEC) plot of purified recombinant FLAG-VMP1. (**B**) Coomassie blue-stained SDS-PAGE showing VMP1 from the indicated SEC elution volumes. (**C**) Immunoblot analysis of VMP1 separated by SDS-PAGE. (**D**) Immunoblot analysis of VMP1 separated by native gel electrophoresis. (**E**) Immunoprecipitation analysis to detect the interaction of VMP1 with itself or RFP. Consistent results were obtained from at least two independent experiments.

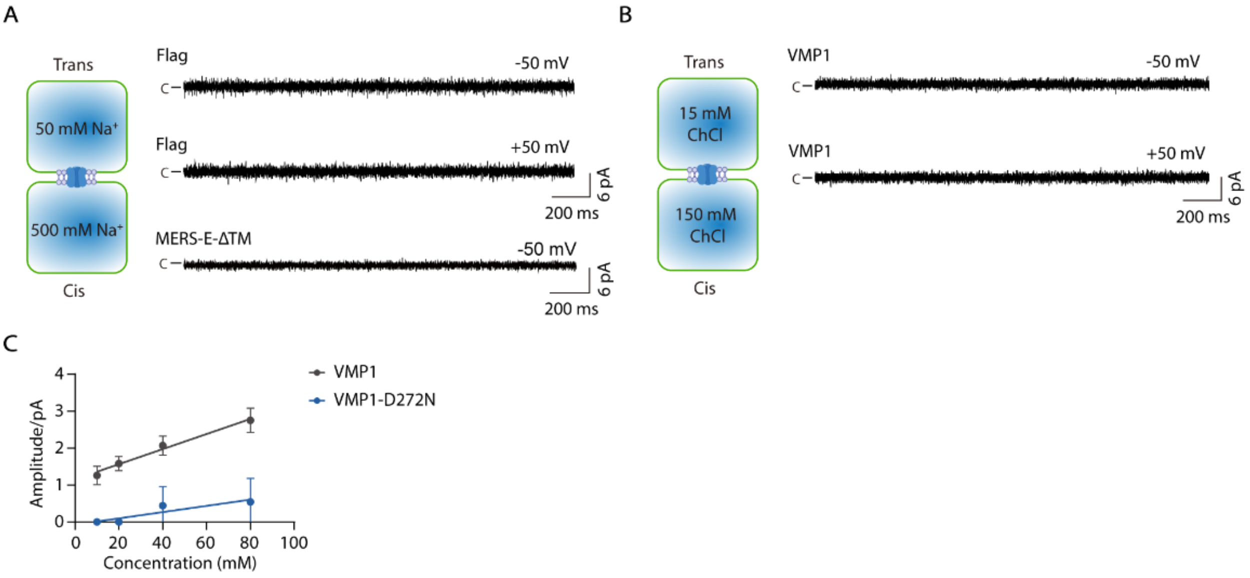

Fig. S7. **Characterization of the ion channel activity of VMP1**. (**A**) Single-channel current recording of purified Flag peptide and truncated membrane protein (MERS-E-ΔTM) in a 500 mM: 50 mM NaCl solution. The Flag peptide or MERS-E-ΔTM was added to the *Cis* side. C denotes the closed state. (**B**) Single-channel current recording of VMP1 in a 150 mM: 15 mM ChCl solution. VMP1 was added to the *Cis* side. (**C**) Concentration-amplitude curve of VMP1 and VMP1-D272N in Ca^{2+} titration experiments. Data represent the mean ± s.e.m. n indicates the number of independent experiments ($n \geq 3$).

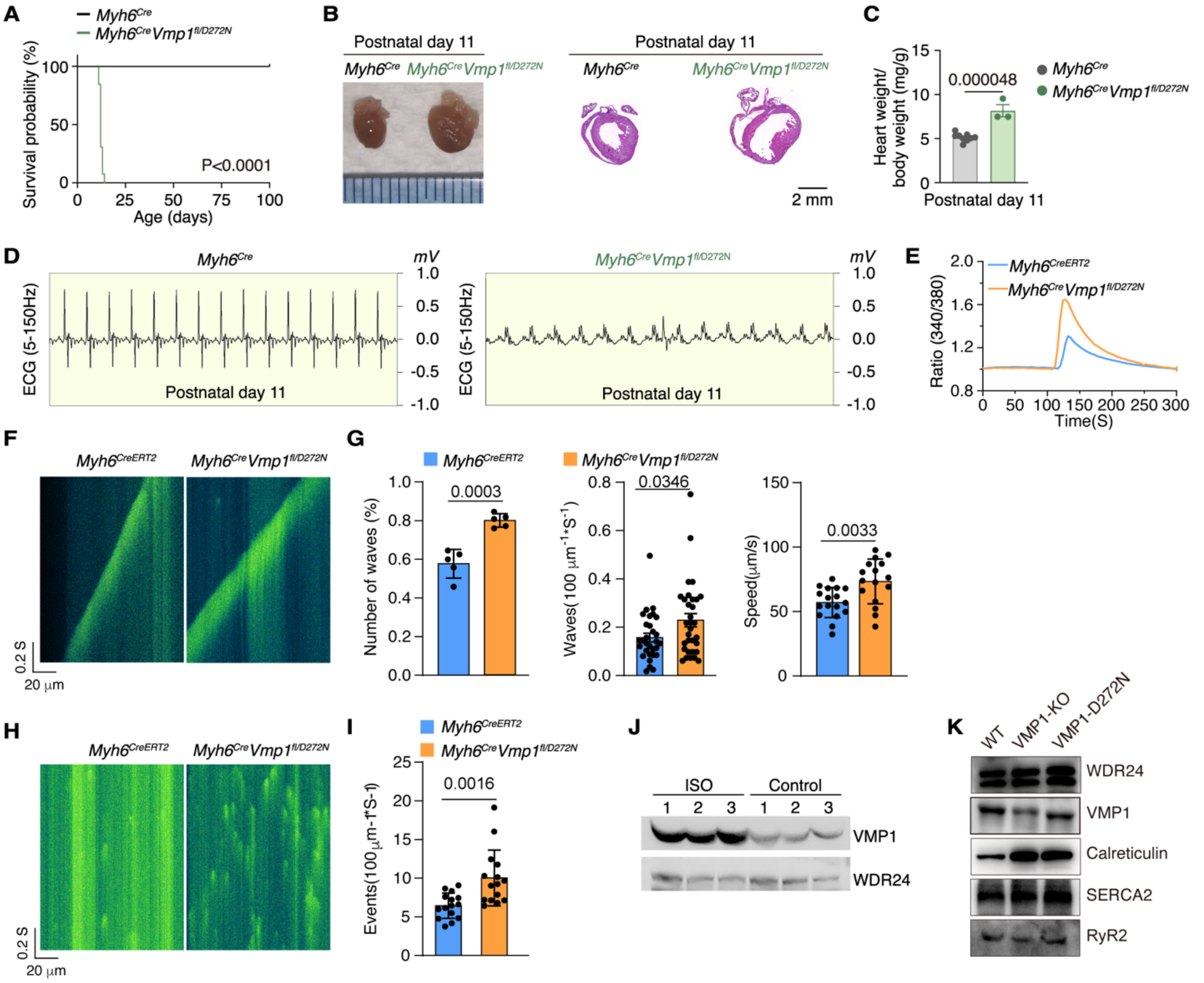

Fig. S8. Characterization of the cardiomyocyte-specific VMP1-D272N mouse model and VMP1 expression of heart failure mice. (**A**) Survival curve of control and *Myh6*^{Cre}*Vmp1*$^{fl/D272N}$ mice (n = 13 mice per group). (**B**) Representative images of hearts and H&E staining of longitudinal heart sections from control and *Myh6*^{Cre}*Vmp1*$^{fl/D272N}$ mice at postnatal day 11. **(C)** Heart weight/body weight ratios in control mice (n = 9) and *Myh6*^{CreERT2}*Vmp1*$^{fl/D272N}$ mice (n = 3) at postnatal day 11. The control group data are shared with Fig. 1G. (**D**) Representative ECG recordings from control and *Myh6*^{Cre}*Vmp1*$^{fl/D272N}$ mice at postnatal day 11. (**E**) Representative average traces of Ca^{2+} imaging of cardiomyocytes isolated from control and *Myh6*^{CreERT2}*Vmp1*$^{fl/D272N}$ mice at 21 days post-tamoxifen injection. (**F-I**) Ca^{2+} waves and sparks were analyzed in cardiomyocytes isolated from control and *Myh6*^{CreERT2}*Vmp1*$^{fl/D272N}$ mice at 21 days post-tamoxifen injection. Representative fluorescence surface plots depicting spontaneous Ca^{2+} waves in the indicated genotypes (**F**). Quantitative analysis of percentage (n = 5), occurrence frequency (n ≥ 30), and speed (n ≥ 16) of spontaneous Ca^{2+} waves in cardiomyocytes isolated from control and *Myh6*^{CreERT2}*Vmp1*$^{fl/D272N}$ mice. (**G**) Representative fluorescence surface plot of spontaneous Ca^{2+} sparks in control and *Myh6*^{CreERT2}*Vmp1*$^{fl/D272N}$ cardiomyocytes. (**H**) Scatter plots quantifying Ca^{2+} spark events in the indicated genotypes (I). (**J**) VMP1 protein expression levels in heart tissues from isoproterenol (ISO)-induced heart failure mice and control mice. (**K**) Western

blot analysis of key sarcoplasmic reticulum (SR) calcium-handling proteins in ventricular cardiomyocytes isolated from wild-type (WT), VMP1 knockout (KO), and VMP1-D272N mice. Protein lysates were probed for RyR2, SERCA2a, and the luminal Ca^{2+}-binding chaperone calreticulin. Data represent the mean ± s.e.m. n indicates the number of mice. Exact P values were determined by log-rank (Mantel-Cox) test in (A) and two-tailed unpaired Student's t-test in (C, G and I).
